# A unified digital twin framework for predicting therapeutic response to central nervous system infections by pathogenic free-living amoebae

**DOI:** 10.1007/s00436-026-08674-6

**Published:** 2026-04-22

**Authors:** Ruqaiyyah Siddiqui, Sutherland K Maciver, David Lloyd, Naveed Ahmed Khan

**Affiliations:** 1 Institute of Biological Chemistry, Biophysics and Bioengineering, Heriot- Watt University Edinburgh, Edinburgh, EH14 4AS UK; 2 Microbiota Research Center, Istinye University, Istanbul, 34010 Turkey; 3 Institute for Neuroscience and Cardiovascular Research, Edinburgh Medical School, University of Edinburgh, Edinburgh, UK; 4 Microbiology Research, School of Biosciences, Cardiff University, P. O. Box 915, Cardiff, CF10 3TL UK; 5 School of Science, College of Science and Engineering, University of Derby, Derby, DE22 1GB UK

**Keywords:** Protozoa, Protists, Digital twin, Predictive tools, Precision medicine

## Abstract

Free-living amoebae such as *Acanthamoeba*, *Balamuthia mandrillaris*, and *Naegleria fowleri* cause lethal infections of the central nervous system, with mortality rates exceeding 90%, despite intensive therapy. These infections remain among the most challenging in clinical practice because therapeutic outcomes are unpredictable and there are no reliable prognostic markers. This article proposes the use of a unified, treatment-centred digital twin framework capable of integrating molecular, pharmacological, immunological, and imaging data to simulate patient-specific responses in real time. By continuously assimilating clinical and biological information, the model forecasts lesion regression, survival probability, and toxicity thresholds under different therapeutic regimens. In contrast to static empirical approaches, this adaptive system can support dose adjustment, predict failure earlier than imaging alone, and test drug combinations virtually before administration. Such a paradigm could transform management of amoebic encephalitis from empirical to predictive medicine, providing a transferable foundation for other neglected central nervous system infections.

## Introduction

Encephalitis caused by *Acanthamoeba* spp., *B. mandrillaris*, and *Naegleria fowleri* represent some of the most devastating parasitic diseases of the central nervous system (CNS) (Berger 2022; Mungroo et al. 2022; Visvesvara et al. 2007). Despite decades of investigation, mortality remains high (Cope et al. 2019; Gharpure et al. 2021; Damhorst et al. 2022). A delayed diagnosis and absence of effective treatment results in poor prognosis (Spottiswoode et al. 2024). The heterogeneity of clinical response, the absence of robust biomarkers, and the inability to predict patient outcomes, suggest the need for a new paradigm that unites biological insight with computational prediction.

In recent years, the concept of a digital twin, i.e., an adaptive computational model that continuously mirrors the physiological and pathological state of an individual, has emerged as a powerful tool in biomedical research (Grieves and Vickers 2016; Katsoulakis et al. 2024). By integrating mechanistic understanding with real-time patient data, digital twins enable dynamic simulation of disease progression and treatment response. When applied to rare, fatal infections such as amoebic encephalitis, a digital twin could provide the interpretability of a mechanistic model with the adaptability of data-driven learning, allowing therapy to be optimised for each patient rather than extrapolated from isolated cases (Li et al. 2025; Sadée et al. 2025; Rahimi et al. 2024). To date, digital twin research has largely focused on oncology, cardiology, and metabolic disorders, with minimal application to infectious or neuroinflammatory diseases (Li et al. 2025; Sadée et al. 2025).

## Rationale for a treatment-centric digital twin

The rationale for developing a digital twin for amoebic encephalitis arises from the gap between the complexity of these infections and the simplicity of their current management. Despite molecular evidence describing host-pathogen interactions, drug mechanisms, and immune dysregulation, clinical care remains reactive rather than predictive (Lorenzo-Morales et al. 2013; Ong et al. 2017). Patients are treated with empirical multidrug regimens that are not formally evaluated in trials (Debnath 2021), doses are adjusted by clinical intuition, and response is judged retrospectively by imaging or neurological improvement. The result is a cycle of therapeutic uncertainty in which neither success nor failure can be anticipated early enough to alter outcomes.

The limitations are structural rather than scientific. Data on drug pharmacokinetics, host immune signatures, and pathogen dynamics exist in silos that rarely intersect in clinical decision-making (Król-Turmińska and Olender 2017; Debnath 2021). For example, knowledge of *Naegleria fowleri*’s rapid disease course is seldom integrated with real-time pharmacokinetic data to forecast the therapeutic window, while the resilience of *Acanthamoeba* cysts is not quantitatively linked to dosing duration or immune modulation (Ong et al. 2017; Anwar et al. 2018). Similarly, immune response parameters such as IL-6 and IL-8 levels, which reflect inflammatory burden, are seldom used to predict treatment response despite being measurable.

A treatment-centric digital twin aims to unify these data within a dynamic computational model that evolves alongside the patient (Sadée et al. 2025; Rahimi et al. 2024; Vallée 2024). In contrast to static predictors, the twin functions as a continuously learning system, drawing on mechanistic biology, pharmacology, and clinical observation. By simulating how a given patient’s immune state, pathogen load, and drug exposure interact over time, it can identify early inflection points in therapy, escalation, or de-escalation may improve survival (Li et al. 2025; Wang et al. 2024). This paradigm reflects a transition from empirical to model-informed precision medicine, allowing clinicians to intervene before clinical deterioration rather than in response to it. Herein we present a conceptual framework intended to guide future development rather than a fully implemented model. Furthermore, the framework contains elements that can be developed using currently available data as well as components that will require further evidence before they can be implemented.

The urgency of this approach reflects the biology of the pathogens themselves. All three free-living amoebae operate at the edge of therapeutic reversibility. In *Naegleria* infection, fulminant inflammation and necrosis leave a therapeutic window of hours (Ong et al. 2017; Gharpure et al. 2021). In *Balamuthia*, the slow, granulomatous course allows prolonged therapy but unpredictable relapse (Kulsoom et al. 2014; Siddiqui and Khan 2008); and in *Acanthamoeba*, the organism’s ability to encyst provides sanctuary from most drugs (Anwar et al. 2018). A single, coherent model that integrates these temporal and mechanistic variables into real-time therapeutic forecasts could shift management from reactive salvage to proactive control. In doing so, the digital twin also creates a scientific infrastructure for learning from every treated case, transforming each patient into a data point that refines the model.

## Clinical and biological context

**Fig. 1:**
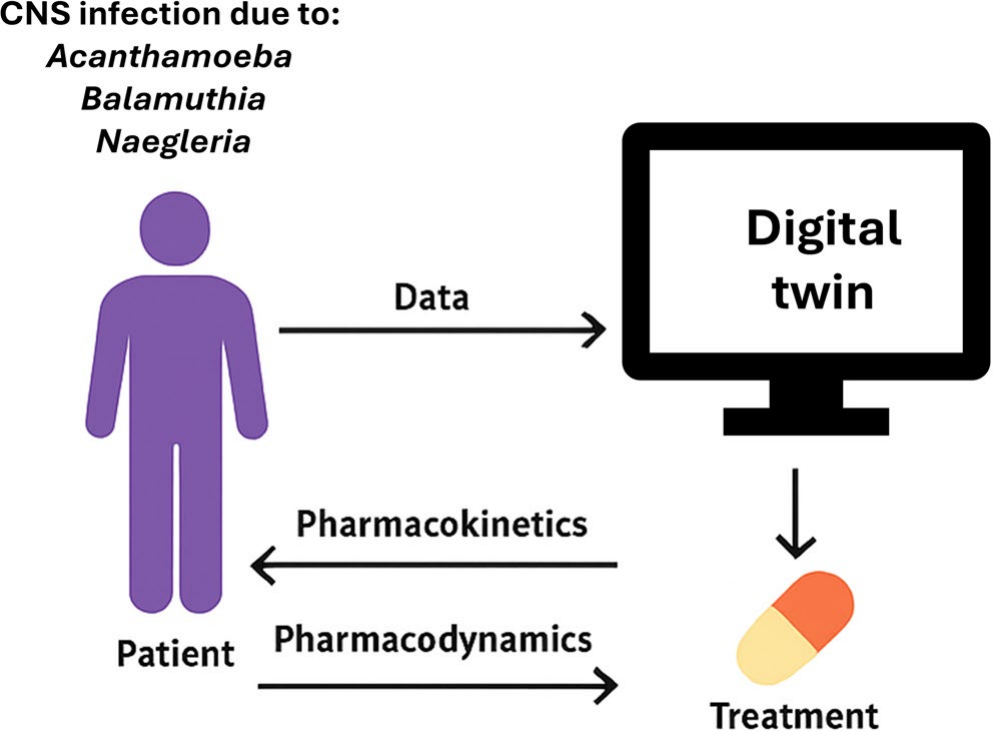
Conceptual representation of a unified digital twin framework for amoebic encephalitis integrating host-pathogen biology, pharmacokinetics, and clinical data to forecast treatment response and optimise therapy

Free-living amoebae vary in tempo and presentation but converge on a common pathophysiological framework. *Acanthamoeba* and *Balamuthia mandrillaris* cause granulomatous amoebic encephalitis, typically evolving over weeks or months, while *Naegleria fowleri* produces primary amoebic meningoencephalitis that progresses within days (Visvesvara et al. 2007; Berger 2022; Gharpure et al. 2021; Damhorst et al. 2022). The three organisms differ in their routes of entry, cutaneous, respiratory, or olfactory but share the ability to traverse epithelial barriers, invade the bloodstream, cross the blood-brain barrier, and induce severe inflammation and necrosis of neural tissue (Lorenzo-Morales et al. 2013; Rodriguez-Anaya et al. 2021; Otero-Ruiz et al. 2023). Each infection is characterised by extensive protease and phospholipase secretion, host-cell lysis, and cytokine release (Visvesvara et al. 2007; Bhosale and Parija 2021; Mungroo et al. 2022). Despite the use of combinations of azoles, miltefosine, pentamidine, amphotericin B, and macrolides, outcomes are rarely favourable, largely because therapeutic success depends on individual variations in immune response, drug penetration, and tolerance to toxicity (Debnath 2021). This biological and therapeutic complexity provides an ideal use case for a digital twin model that can integrate diverse data streams into coherent, patient-specific predictions (Fig. 1; Table 1). Diagnostic uncertainty, particularly in early or atypical presentations, is recognised and the digital twin is intended to incorporate evolving clinical information rather than depend solely on an initial diagnosis.

**Table 1:** Comparative features of free-living amoebic encephalitis

Characteristic	Acanthamoeba	Balamuthia mandrillaris	Naegleria fowleri
Clinical form	Granulomatous amoebic encephalitis	Granulomatous amoebic encephalitis	Primary amoebic meningoencephalitis
Disease tempo	Chronic (weeks–months)	Sub-acute (weeks)	Acute (days)
Entry route	Cutaneous or nasal	Cutaneous or nasal	Nasal (olfactory neuroepithelium)
Immune profile	Th1-dominant, granulomatous	Mixed granulomatous-neutrophilic	Neutrophilic, hyper-inflammatory
Key virulence factors	Mannose-binding protein, serine and cysteine proteases, cyst formation	Proteases, phospholipases, galactose-binding protein	Nfa1 adhesin, phospholipases, elastase
Drug regimen	Azoles, miltefosine, pentamidine	Multidrug combinations, prolonged therapy	Amphotericin B, azoles, rifampicin, miltefosine
Mortality	> 90%	> 90%	> 95%

## Unified digital twin framework

The unified digital twin for amoebic encephalitis is conceived as a modular yet continuous system that mirrors the biological and therapeutic dynamics of infection within the central nervous system (Li et al. 2025; Rahimi et al. 2024; De Domenico et al. 2025; Wang et al. 2024). At its core lies a mechanistic scaffold linking three interacting processes: pathogen kinetics, host immune response, and drug behaviour within the brain microenvironment. Each of these processes is represented by a set of differential equations and probabilistic rules that evolve with patient data, generating an ever-updating computational analogue of the disease process. The framework begins with the pathogen-host interface, where molecular mechanisms such as amoebic adhesion, protease secretion, and endothelial injury are represented as parameterised rates influencing tissue invasion (Rodriguez-Anaya et al. 2021; Bhosale and Parija 2021; Otero-Ruiz et al. 2023). These variables are directly informed by experimental data on enzyme kinetics and blood-brain barrier permeability. Of note, behaviour related to the cyst stage should be treated as a biological property, inferred from existing knowledge of the organism rather than as a direct clinical measurement, since cysts are not usually detected in CSF. Cyst to trophozoite transitions can be represented conceptually using time-dependent rates informed by experimental observations of *Acanthamoeba* biology (Siddiqui et al. 2019), with specific mathematical forms to be developed when suitable data become available. The host component will model cytokine production, immune cell recruitment, and tissue inflammation using time-dependent feedback terms that can reflect amplification and resolution phases of the immune response (Niarakis et al. 2024; Wang et al. 2024). Together, these parameters define the infection’s intrinsic tempo and the intensity of the inflammatory reaction (Table 2). Of note, variables representing pathogen burden are also included to reflect how pathogen kinetics are captured within the framework. The equations themselves are not specified at this stage, since their final form will depend on the availability and quality of pathogen, immune, and pharmacokinetic data during future model development.

**Table 2:** Conceptual model parameters linking biological phenomena to computational behaviour within the unified digital twin framework. kₜ represents the disease tempo constant; Bₚ denotes pathogen burden; P₍BBB₎ denotes blood–brain barrier permeability; τ_i_ is the immune activation delay; fₚₖ reflects pharmacokinetic penetration fraction; Eₚ indicates pathogen factors; and H represents the baseline mortality hazard. Values are illustrative and intended to demonstrate how species-specific biological differences can be expressed quantitatively within a shared model topology

Model variable	Conceptual role in digital twin	Biological correlate	Illustrative range / qualitative trend
kₜ	Represents disease tempo and rate of progression	Clinical course from onset to neurological involvement	Low in *Acanthamoeba*, intermediate in *Balamuthia*, high in *Naegleria*
Bₚ	Represents amoebic load within CNS tissue over time	Trophozoite density, inferred lesion burden, PCR signal where available.	Very high in *Naegleria* early; variable but rising in *Acanthamoeba*; fluctuating in *Balamuthia* with granulomatous phases.
P₍BBB₎	Determines drug penetration across the blood–brain barrier	Extent of CNS exposure to therapy	Increases from *Acanthamoeba* to *Naegleria*
τ_i_	Time constant for immune activation	Cytokine and leukocyte response lag	Shortest in *Naegleria*
fₚₖ	Pharmacokinetic penetration fraction	Drug concentration ratio between plasma and CSF	Intermediate in *Balamuthia*, variable among drugs
Eₚ	Pathogen factors	Dormancy and survival potential	High in *Acanthamoeba*, moderate in *Balamuthia*, and *Naegleria*
H	Baseline hazard or mortality parameter	Intrinsic lethality of infection	Highest in *Naegleria*

**Fig. 2:**
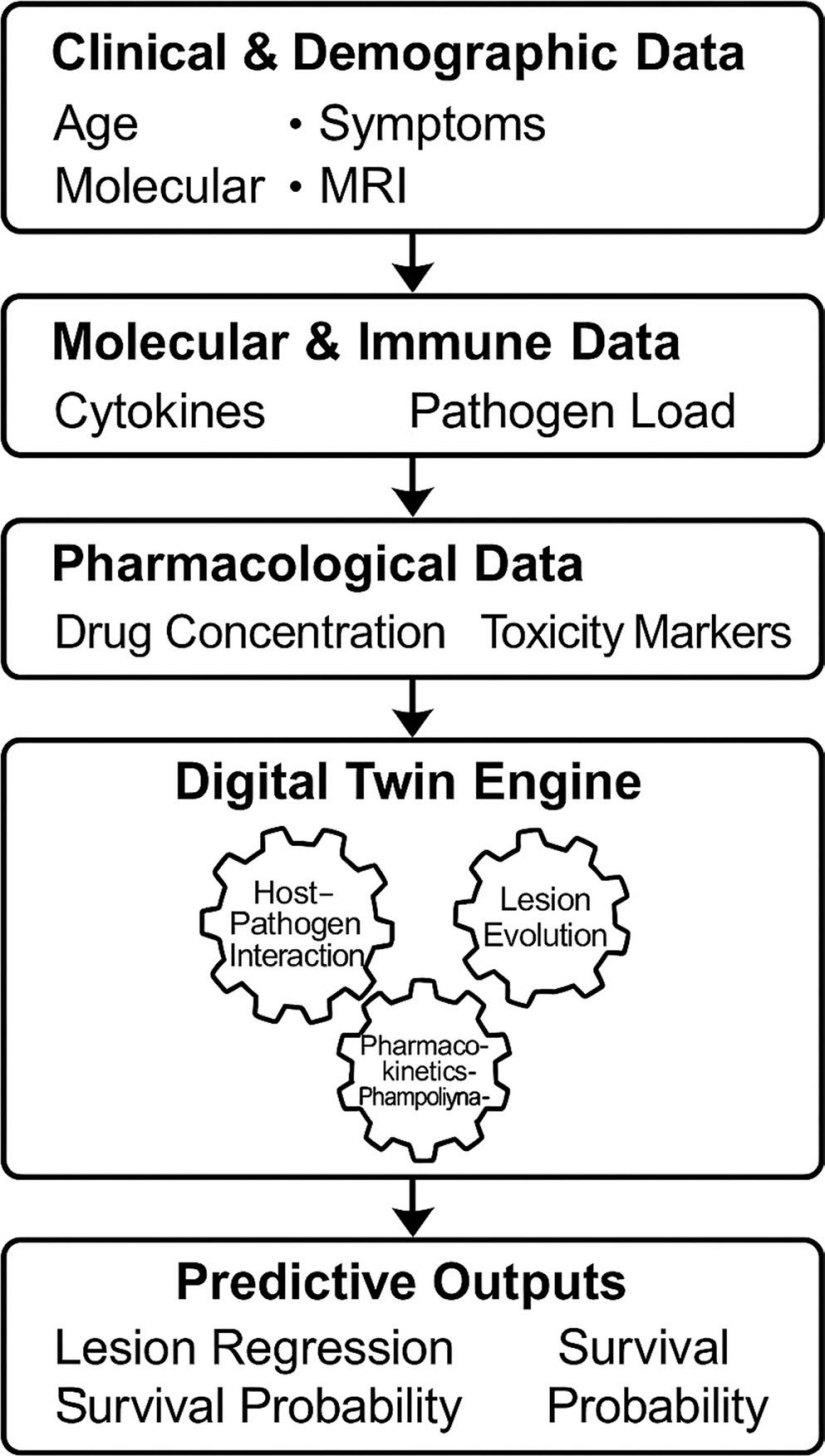
Multidimensional data integration within the unified digital twin enables simulation of disease behaviour and adaptive therapy in real time

Superimposed on this biological substrate is the pharmacokinetic-pharmacodynamic (PK/PD) layer, which tracks drug concentration within plasma, cerebrospinal fluid (CSF), and parenchyma (Coggins and Greenberg 2025; Neves-Zaph and Kaddi 2024). It models diffusion across the blood-brain barrier, drug-target interaction with trophozoites or cysts, and cumulative toxicity within host tissues. Each therapeutic agent is represented by its absorption, distribution, metabolism, and excretion constants, drawn from experimental or clinical data. The PK/PD module interacts with the pathogen–host layer through dynamic coupling: as drug levels rise, amoebic kill rates increase and inflammatory mediators decline, altering both parasite burden and host response (Fig. 2). Examples of parameter sources may be drawn from clinical reports describing therapeutic experience with miltefosine in primary amoebic meningoencephalitis (Cope et al. 2016) and meta-analytic studies characterising CSF cytokine patterns in encephalitis (Soltani Khaboushan et al. 2022), with specific datasets selected according to availability during future model development. A simplified early prototype could be developed by combining basic clinical markers with imaging and pharmacokinetic information, with further components added as additional data become available.

The lesion evolution module bridges molecular events with clinical observables. It converts the combined outputs of the biological and pharmacological layers into macroscopic imaging correlates, such as changes in lesion size or contrast intensity on magnetic resonance imaging (MRI). This layer forms the tangible interface between the computational model and clinical monitoring, enabling direct comparison of simulated and observed disease trajectories (Barbiero et al. 2021; Bhagirath et al. 2024; Coorey et al. 2022). When the model’s predicted lesion volume diverges from measured data, recalibration occurs, ensuring that the twin remains synchronised with the patient’s evolving condition (Laubenbacher et al. 2021; Esposito et al. 2025).

Finally, the outcome layer translates these dynamic interactions into clinically meaningful forecasts: the probability of radiological improvement, predicted time to stabilisation, and likelihood of treatment-limiting toxicity. Improvement probability can be recalculated each time new clinical, imaging, or laboratory information is incorporated, allowing the estimate to evolve with the patient’s course rather than remaining fixed. Because all layers are interconnected, feedback from any domain including new imaging, laboratory results, or pharmacokinetic data propagates throughout the system, refining every other variable. Nonetheless, these references to survival probability, lesion behaviour, and toxicity thresholds reflect conceptual projections within the proposed model and will require future validation before clinical application.

**Fig. 3:**
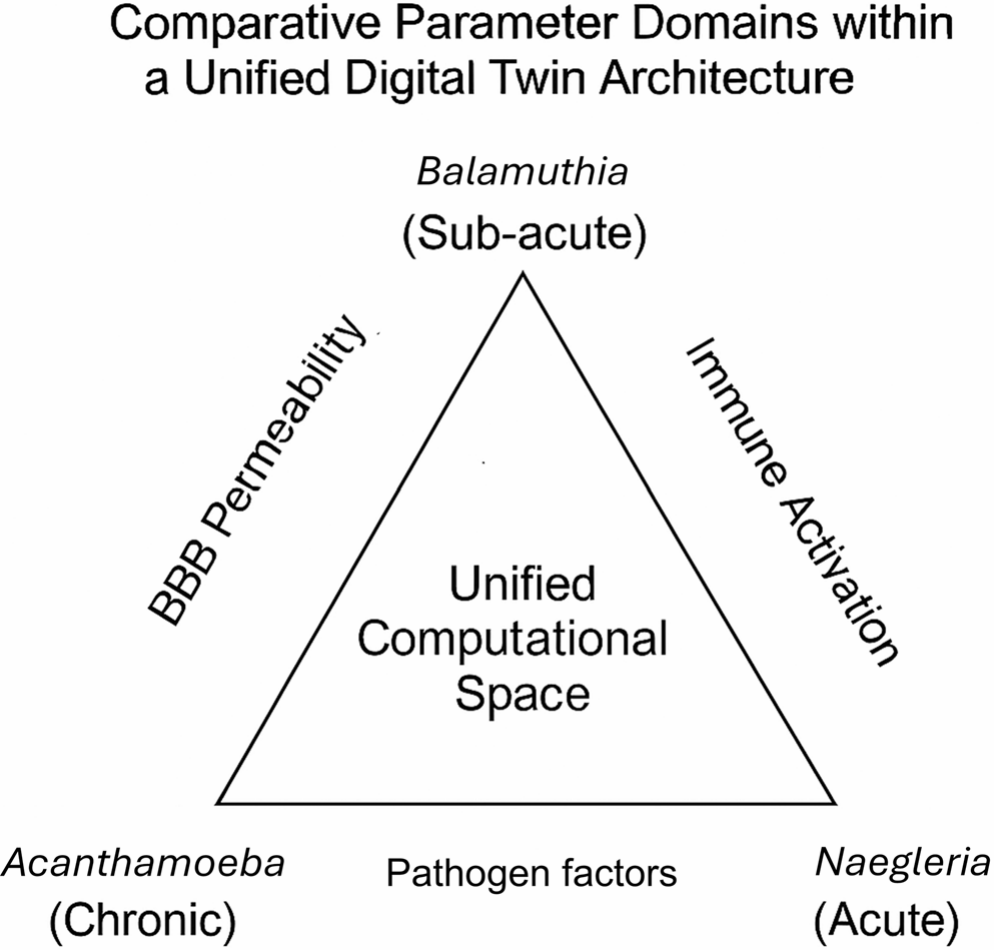
Species-specific parameter domains within a single computational topology. Variation in disease tempo, immune dynamics, and drug permeability allows the twin to simulate chronic, sub-acute, and fulminant amoebic encephalitis using a unified architecture

Importantly, this unified structure accommodates pathogen diversity not by creating separate models but by allowing quantitative modulation of shared parameters. *Acanthamoeba* occupies the slow-tempo, high-encystment region of parameter space, *Balamuthia* represents an intermediate tempo with mixed immune activation, and *Naegleria* lies at the rapid-progression extreme characterised by explosive inflammation and minimal encystment (Lorenzo-Morales et al. 2013; Rodriguez-Anaya et al. 2021; Otero-Ruiz et al. 2023). By spanning this spectrum within one computational topology, the digital twin becomes both species-specific and generalisable (Fig. 3). Differences in inflammatory behaviour across species can be reflected conceptually by using rapid, intense inflammatory rates for *Naegleria fowleri* and slower, more prolonged rates for the granulomatous inflammation seen in *Balamuthia mandrillaris*, with specific values to be defined during later model development.

The result is a virtual clinical environment where therapies can be tested in silico before they are administered, risks quantified before toxicity occurs, and outcomes anticipated before irreversible damage develops (Esposito et al. 2025; Nadeem et al. 2025). Each new case will contribute to the refinement of model parameters, gradually transforming isolated experiences into a collective learning framework. In this sense, the unified digital twin is not merely a simulation of a single patient but an evolving embodiment of collective biological understanding, one that grows more accurate with every life it models.

## Clinical utility and translational applications

The unified digital twin may offer several translational advantages (Barbiero et al. 2021). It allows adaptive optimisation of therapy by forecasting outcomes under different dosing or drug combinations before they are applied clinically (Ahmed et al. 2023). It provides early indicators of response or failure by detecting divergence between simulated and observed lesion trajectories, potentially weeks before visible clinical improvement. It can also be used as a virtual testing ground for repurposed drugs, simulating pharmacokinetic and pharmacodynamic interactions to identify promising candidates for further study. Over time, aggregated data from individual twins could be integrated into a global learning system that continuously refines parameter estimates and predictive accuracy, creating a self-improving model for rare central nervous system infections.

A hypothetical clinical scenario illustrates its utility. A teenage patient diagnosed with *Balamuthia mandrillaris* encephalitis begins therapy with miltefosine, fluconazole, and pentamidine. Her baseline MRI and pharmacokinetic data initialise the twin. After ten days, cytokine levels fall but lesion size remains unchanged. The twin predicts a 60% probability of improvement if therapy continues unchanged, but 80% if miltefosine exposure increases by 20% without exceeding hepatotoxic thresholds. The clinical team adjusts the dose accordingly, and subsequent imaging confirms improvement consistent with model predictions. This iterative, data-driven feedback exemplifies how the digital twin can guide real-time therapeutic decision-making in an otherwise unpredictable disease.

## Integration with broader computational medicine

The treatment-centric digital twin for amoebic encephalitis aligns with emerging trends in precision medicine that integrate mechanistic modelling with artificial intelligence. However, applications of this approach to infectious diseases, particularly encephalitis remains virtually unexplored. Unlike purely statistical predictors, the twin is anchored in biological realism, capturing measurable processes such as blood-brain barrier permeability and immune feedback. Unlike static mechanistic models, it evolves continuously as new patient data become available. This combination of interpretability and adaptability ensures that predictions remain both physiologically meaningful and clinically actionable. The approach can easily be extended to other rare CNS infections, including fungal and helminthic diseases, where data scarcity and heterogeneity preclude conventional clinical trials. Moreover, federated learning architectures could allow hospitals worldwide to contribute anonymised patient data, improving predictive accuracy while maintaining privacy and data security.

## Challenges and future prospects

Several challenges remain before clinical deployment. Data sparsity is inherent to rare infections, necessitating the use of strong priors and uncertainty quantification. Validation of the model against prospective clinical outcomes will be critical to ensure reliability and ethical acceptability (Bruynseels et al. 2018). The interpretability of outputs must remain central to avoid over-reliance on algorithmic recommendations. Integrating such a system into clinical decision-making will require interdisciplinary governance involving clinicians, modellers, ethicists, and regulatory bodies. Future development should focus on creating multicentre registries, integrating transcriptomic and metabolomic data to refine immune and metabolic pathways, and developing intuitive clinician-facing interfaces that visualise model outputs transparently. The conceptual nature of the framework reflects the need for staged development, with translational progress expected to follow as suitable clinical and experimental datasets accumulate. Parameter estimation will depend on gradual incorporation of published evidence, clinical observations, and experimental findings as they become available, with refinement occurring over time as additional cases inform the framework. Approaches for handling uncertainty and informing model refinement will be selected during later stages of development, guided by the availability and suitability of clinical, experimental, and published data. The ultimate goal is a bedside system capable of real-time simulation of treatment trajectories, helping physicians adapt therapy before irreversible damage occurs.

## Conclusion

Amoebic encephalitis represents a frontier of unmet medical need where empirical therapy has reached its limits. A unified, treatment-focused digital twin offers a pathway to predictive, adaptive, and personalised care. By embedding shared biological mechanisms and therapeutic variables of *Acanthamoeba*, *Balamuthia*, and *Naegleria* within a single computational framework, the model transcends species boundaries and captures the essential logic of infection dynamics and treatment response. This approach not only holds promise for improving survival in these neglected diseases but also establishes a template for applying digital twin technology to other rare and fatal infections of the central nervous system.

## Data Availability

Not applicable.
